# Office Hysteroscopic Laser Enucleation of Submucous Myomas without Mass Extraction: A Case Series Study

**DOI:** 10.1155/2015/905204

**Published:** 2015-05-18

**Authors:** Sergio Haimovich, Maite López-Yarto, Julio Urresta Ávila, Alejandro Saavedra Tascón, José L. Hernández, Ramón Carreras Collado

**Affiliations:** ^1^Department of Obstetrics and Gynecology, Hospital del Mar, Autonomous University of Barcelona, Barcelona, Spain; ^2^International Master in Reproductive Medicine, Autonomous University of Barcelona, Barcelona, Spain

## Abstract

*Background and Objectives*. A new two-step hysteroscopic myomectomy carried out in the office setting and without anesthesia was feasible for the excision of submucous myomas. The objective of this study was to assess whether removal of submucous myomas from the uterine cavity after hysteroscopic laser enucleation is necessary. *Methods*. Between June 2009 and April 2013, all outpatients with symptomatic myomatosis (bleeding, pelvic pain, and infertility) assessed ultrasonographically were eligible to participate in a prospective study. All patients underwent office hysteroscopic enucleation of submucous myomas. Enucleated myomas were left in the uterine cavity. Neither anesthesia nor antibiotic prophylaxis was used. *Results*. Sixty-one women (mean age: 47.3 years) were included. Regardless of hysteroscopic localization and grading, all myomas were enucleated. The mean (standard deviation, SD) diameter of the myoma as measured by the ultrasound scan was 22.6 (8.5) mm. In 29 cases (47.5%), the diameter of the resected myoma was >20 mm and in 10 cases (16.4%) >30 mm. After a mean follow-up of 68.2 (16.5) days, none of the patients showed a residual myoma inside the uterine cavity. *Conclusions*. The present results indicate that leaving laser-enucleated submucous myoma in the uterine cavity is a feasible and safe therapeutic option.

## 1. Introduction

Uterine myomas or fibroids are the most common benign tumors of the female genital tract and are estimated to occur in about 30% of women by the age of 35 and in about 70–80% over the age of 50 [1]. Uterine myomas form the most frequent indication for hysterectomy [2]. Although most women are asymptomatic at least one out of four present clinical symptoms [3, 4], resulting in 3–5% of gynecologic consultations [5]. Depending on their location in the uterus, they may be subserous, intramural, or submucous. Also, the localization appears to be an important factor in determining the frequency and severity of clinical symptoms. Submucous myomas account for 5.5% to 16.6% of all uterine myomas [6] and are a common structural cause of abnormal menstrual bleeding, pelvis pain, infertility, and other symptoms [7, 8].

Hysteroscopic removal is the most effective treatment [9, 10] and is the standard minimally invasive surgical procedure for treating submucous fibroids. Uterine bleeding and infertility are the most frequent indications for surgical excision of the tumor. Hysteroscopic myomectomy was first performed in 1976 and since then it has become a safer and more effective procedure after improvements in endoscopic technology and instrumentation [11, 12]. The choice of the technique mostly depends on the intramural extension of the fibroid, as well as the experience of the surgeon and the available equipment. The separation into three grades according to the size of the myoma and the proportion of myoma protruding into the uterine cavity has been shown to be efficient at discriminating the complexity of hysteroscopic myomectomies [5, 13].

Most techniques aim at the transformation of an intramural fibroid into a totally intracavitary lesion, thus avoiding a deep cut into the myometrium. “Resectoscopic slicing” still represents the “gold standard” technique for treating fibroids G0. Other effective techniques, such as ablation by neodymium-yttrium-aluminum-garnet laser, morcellation [14, 15], and office myomectomy, have been proposed, with a recent increase in the use of morcellators in hysteroscopy. Both resectoscopy and the use of hysteroscopic morcellator as an alternative to conventional resectoscopy are the techniques used for removal of submucosal myomas. The morcellator technique has the advantage that tissue fragments can be removed easily by means of suction through the instrument and are thereafter still available for histologic analysis [5].

Recently, our group developed a two-step office hysteroscopic resection of intracavitary G1 or G2 submucous myomas as an outpatient procedure without anesthesia [16]. This procedure using an only 4.3 mm hysteroscope involves leaving the enucleated myoma inside the uterine cavity. After successful results of a pilot study [16], a prospective study was designed to assess whether or not removal of submucous myomas from the uterine cavity after hysteroscopic laser enucleation is necessary.

## 2. Materials and Methods

### 2.1. Patients

Between June 2009 and April 2013, a prospective study was performed at the outpatient hysteroscopic unit of the Service of Obstetrics and Gynecology of an acute-care teaching hospital in Barcelona, Spain. Eligible women were those of reproductive age with symptomatic myomatosis, including uterine bleeding, pain, and infertility, and sonographically diagnosed as single G0, G1, or G2 myoma. All participants underwent hysteroscopic laser enucleation of the tumor, the removal of which from the uterine cavity was not possible due to the size of the mass. Patients who did not meet the inclusion criteria were excluded from the study as were patients in whom myomas could not be completely enucleated, those with a histological diagnosis other than fibroids and patients lost to follow-up ultrasound studies. The study was conducted according to the principles of Helsinki declaration (1975), and all participants signed written informed consent.

### 2.2. Transvaginal Ultrasound Scanning

During the first half of the menstrual cycle, transvaginal ultrasound examination was performed in all patients to assess the characteristics of the submucous myoma and to exclude other coexistent lesions. The size of the myoma was defined by means of the three diameters as measured in the longitudinal, anteroposterior, and transversal planes. Fibroids were classified according to the classification system adopted by the European Society for Gynecological Endoscopy (ESGE) based on the degree of penetration of the submucous myoma in the endometrium (G0 within total intracavitary development, G1 with mostly [>50%] intracavitary development, and G2 with >50% intramural development) [13]. Ultrasonographic studies were performed by trained sonographers with a 5.0–9.0 MHz multifrequency transvaginal probe (Voluson 730 Expert, General Electric's Medical Systems, Kretztechnik, Zipf, Austria).

### 2.3. Hysteroscopic Procedure

Patients were prepared with desogestrel 75 *μ*g/day, for at least 6 weeks before the procedure to achieve endometrial atrophy [17]. The night before the procedure, intravaginal misoprostol 200 *μ*g was indicated (400 *μ*g in nulliparous women). All patients received oral diazepam, 10 mg, and ibuprofen, 600 mg, 30 minutes before the office procedure. Prophylactic antibiotics were not given.

Hysteroscopy procedures were performed by the same examiner (S.H.) with a 4 mm continuous flow office hysteroscope (Bettocchi Office Hysteroscope size 4, Karl Storz, Tuttlingen, Germany) with a 2.9 mm rod lens optical system. Distension of the uterine cavity was performed with saline solution; intrauterine pressure was controlled by a system of pumps. The office procedure was carried out without anesthesia, speculum, or Pozzi tenaculum; fibrosis was identified by localization and size and classified according to ESGE criteria [13]. Myoma resection was performed with high-power 980 nM diode laser using a 1000-micron diamond probe (BioLitec, Germany). According to the degree of penetration, resections were performed differently. When myoma was G0, the pedicle was sectioned; when myoma was G1, the base was resected and if necessary, like in G2 myomas, the mass was enucleated by the two-step technique previously described [16]. All enucleated myomas were left free in the uterine cavity, tissue sampling for histological diagnosis was taken, and the characteristics of uterine cavity were evaluated. At the end of the procedure, patients were observed for 20 minutes before leaving the hysteroscopy unit.

### 2.4. Follow-Up

A second transvaginal ultrasound examination was performed within 60 to 90 days after the hysteroscopic procedure to assess the presence or absence of myoma inside the uterine cavity. The degree of satisfaction and presence of complication was evaluated within 10 to 15 days after ultrasound studies through a telephone call. The level of satisfaction was quantified using a 5-point Likert verbal scale from 0 being not at all satisfied to 5 being very satisfied.

The absence of myoma on the follow-up ultrasound examination was the outcome variable of the study.

### 2.5. Statistical Analysis

Categorical variables are expressed as frequencies and percentages and quantitative variables as mean and standard deviation (SD) or median and interquartile range (IQR) (25th–75th percentile). Descriptive statistics are presented. The SPSS (version 18.0 for Windows) (IBM Corp.) was used for data analyses.

## 3. Results

During the study period, a total of 61 women fulfilled the inclusion criteria and were included in the study. The mean age was 47.3 (8.5) years. Six patients were nulliparous. Bleeding was the most frequent symptom. The characteristics of patients are shown in Table 1. The mean diameter of the myoma as measured by the ultrasound examination was 22.6 (8.5) mm, and most tumors were found in the anterior and lateral uterine walls. In 47.5% of patients, the size of the myoma was ≥20 mm and in 16.4% it was larger than 30 mm. In relation to hysteroscopic grading, G1 tumors accounted for 51.1% of cases and G2 accounted for 16.4% (Table 2).

In none of the patients, the echographic check-up carried out within 60 to 90 days after the procedure revealed the presence of a myoma in the uterine cavity. The mean follow-up was 68.2 (16.5) days. Most patients were asymptomatic. Thirteen women reported bleeding and three reported discomfort which were considered as mild. The overall degree of satisfaction was very high, with a median of 4.55 (Table 2).

## 4. Discussion

Results of the present prospective study show that ambulatory hysteroscopic removal of submucous myomas carried out without anesthesia and leaving free the mass in the uterine cavity after enucleation is a feasible and safe procedure. In all patients, the absence of myoma was confirmed on the follow-up ultrasound examination. At follow-up, most patients were asymptomatic and had a high degree of satisfaction with the procedure. Until today, many hysteroscopy myomectomy techniques have been described, including different ways for the extraction of the myoma (slicing, morcellation, vaporization, etc.) from the uterine cavity. The myoma enucleation technique used by our group uses a 4.3 mm hysteroscope and most of the enucleated fibroids could not be extracted out through the endocervical channel. As far as we are aware, this is the first report of the option of leaving the enucleated mass in the uterine cavity.

No cases of painful myoma expulsion were reported. The enucleated fibroid has no vascularization and a necrosis process begins, reaching a stage of a smooth mass that can be pushed out without causing major discomfort. The process of expulsion often takes place during the menstruation or, as some of the patients reported, in a spontaneous way. On the other hand, there is low risk of malignancy. The outcome of occult leiomyosarcoma after surgery for presumed uterine fibroids is a controversial issue. The probability of finding a leiomyosarcoma in a patient with a tentative diagnosis of myoma is low (0.23–0.49%), being more frequent in postmenopausal women, intramural rather than submucous lesions [18, 19]. In a systematic review to assess whether morcellation of occult leiomyosarcomas during surgery to treat presumed myomas may substantially worsen patient outcome, no reliable evidence that morcellation substantially results in tumor upstaging was found [20]. There is no indication for myomectomy in women in the reproductive age with asymptomatic fibroids. In those women with a growing or symptomatic fibroid (e.g., bleeding), malignancy should be suspected and the treatment approach will be different.

The hysteroscopic surgical techniques achieve complete removal of the myoma only in those cases in which the fibroid mass is mostly inside the uterine cavity (G0 and G1), and are based on the progressive extraction of small fibroid tissue pieces. The most frequently used technique is the resectoscopy, based on repeated slices extraction. Although no cases of transtubal dissemination of malignant tissue into the abdominal cavity have ever been reported there is a hypothetical risk [21, 22].

Recently a new miniresectoscope was introduced for office setting; this new devise allows treating fibroids without anesthesia. Due to the small diameter, the slice extraction process and the achievement of a complete surgery take longer and, in case of deep myoma, an optimal surgery is not always achieved because of the perforation risk. In 2009 Bettocchi et al. described a two-step technique for deep myomas in which a first step was performed in office using Versapoint in order to free the fibroid from the pseudocapsule and the second step was performed in surgery room by resectoscopy [23].

All the office myomectomy techniques until now were based on the total extraction of the fibroid's tissue from the uterine cavity.

With the myomectomy technique applied by our group, in contrast to the resectoscopy or the morcellation consisting of sections and repeated destruction of the tumor integrity, surgical planes were respected and a complete enucleation of the fibroid was achieved. We always obtained a tissue sample of the mass for histological diagnosis, this biopsy was performed by laser and this kind of energy cuts but at the same time achieves a tissue cauterization making cell dissemination very unlikely. In our series of a total of 153 hysteroscopic myomectomy enucleation in an office setting, in 61 cases the myoma was left inside the uterine cavity, and no histological diagnosis of malignancy was made in any of the 153 cases. However, if the risk of malignant cell dissemination using laparoscopic techniques is low, it is even lower in the case of hysteroscopic myomectomy. We have been using resectoscopy for submucous fibroids during almost 30 years which continues to be the most used technique for this type of fibroids. In a review of the literature, no case of malignant cell dissemination after resectoscopy for uterine cancer ablation has been published. We cannot state that there is no risk of dissemination during hysteroscopic myomectomy because scientific evidence is lacking, but on the basis of absence of publications and extensive experience during the last 30 years, if there is any risk, it must be very low.

## 5. Conclusions

When mass extraction is not possible, hysteroscopic total enucleation of submucous myoma leaving the mass free inside the uterine cavity is a feasible and safe therapeutic option. The technique can be carried out as an outpatient procedure and without anesthesia. The present results open an effective new treatment modality different from hysteroscopic myomectomy, especially in an office setting, for the management of patients with symptomatic submucous myomas. However, the number of patients included in our series is relatively small and further well-designed studies are needed to confirm the reproducibility of the technique.

## Figures and Tables

**Table 1:** Demographic characteristics of 61 women with symptomatic submucous myoma undergoing office hysteroscopic laser enucleation.

Variable	Data
Age, years, mean (SD)	47.3 (8.5)
Parity, mean (SD)	1.45 (0.98)
Parity, number (%)	
0	13 (21.3)
1	17 (27.8)
2	23 (37.7)
3	8 (13.2)
Indications for surgery	
Bleeding	45 (73.6)
Infertility	8 (13.2)
Pain	8 (13.2)
Size, mm, mean (SD)	22.3 (8.5)

Data presented as absolute numbers and percentages in parentheses unless otherwise stated.

**Table 2:** Results of office hysteroscopic laser enucleation in 61 women with symptomatic submucous myoma.

Variable	Data
Size, mm	
<20	32 (52.4)
20–29	19 (31.1)
>30	10 (16.4)
Hysteroscopic grading	
G0	20 (32.8)
G1	31 (51.1)
G2	10 (16.4)
Location	
Anterior wall	24 (39.3)
Posterior wall	13 (21.3)
Lateral walls	24 (39.3)
Follow-up, days, mean (SD)	68.2 (16.5)
Complications	
None	43 (70.5)
Bleeding	13 (21.3)
Pain	3 (4.9)
Other	2 (3.3)
Degree of satisfaction, median (IQR)	4.5 (4-5)

Data presented as absolute numbers and percentages in parentheses unless otherwise stated.
